# The P2X_7_ loss-of-function Glu496Ala polymorphism affects ex vivo cytokine release and protects against the cytotoxic effects of high ATP-levels

**DOI:** 10.1186/1471-2172-13-64

**Published:** 2012-12-04

**Authors:** Anke Wesselius, Martijn JL Bours, Ilja CW Arts, Esther HE Theunisz, Piet Geusens, Pieter C Dagnelie

**Affiliations:** 1 Department of Epidemiology, Maastricht University, School for Public Health and Primary Care (CAPHRI), Peter Debyeplein 1, Maastricht, MD, 6200, The Netherlands; 2 Department of Epidemiology, Maastricht University, School for Cardiovascular Diseases (CARIM), Maastricht, The Netherlands; 3 Department of Toxicology, Maastricht University Medical Centre School for Public Health and Primary Care (CAPHRI), Maastricht, The Netherlands; 4 Department of Internal Medicine, Subdivision of Rheumatology Maastricht University Medical Center, P. O. Box 5800, Maastricht, 6202 AZ, The Netherlands

**Keywords:** ATP, P2X_7_, Polymorphism, Inflammation

## Abstract

**Background:**

The *P2X*_*7*_ receptor plays an important role in cytokine release during the inflammatory response *in vivo.* Polymorphisms within the *P2X*_*7*_ receptor gene that lead to loss of receptor function may contribute to impaired cytokine release by immune cells. Therefore, we investigated whether a known loss-of-function polymorphism (*Glu496Ala*) in the *P2X*_*7*_ receptor gene leads to alterations in cytokine release in response to ATP.

**Results:**

An ex vivo whole blood model was used to induce an inflammatory reaction with the pro-inflammatory stimuli LPS and PHA (phytohemagglutinin). Blood from n=9 subjects with the Glu496Ala P2X7 SNP (*P2X7MUT*) and n=7 ‘wild-type’ subjects (no P2X7 SNP; *P2X7WT*) was used.

Addition of ATP (0.9-3 mM) to LPS/PHA-stimulated whole blood induced an increase in IL-1β release in *P2X7MUT* subjects, whereas decreased release was observed in *P2X7WT* subjects. Decreased levels of IL-6 and TNF-α in response to ATP were shown in both *P2X7MUT* and *P2X7WT* subjects, which was less pronounced in *P2X7MUT* subjects. ATP at 3 mM also significantly decreased levels of lactate dehydrogenase (LDH) in *P2X7MUT* subjects compared to *P2X7WT* subjects.

**Conclusions:**

The presence of the non-synonymous *Glu496Ala* loss-of-function polymorphism within the *P2X*_*7*_ receptor gene is likely to be of importance in the release of cytokines during inflammation. Furthermore, this study suggests that carriers of the Glu496Ala loss-of-function polymorphism are protected against the cytotoxic effects of high ATP-levels.

## Background

The innate immune system constitutes the first line of defense against anything that compromises tissue homeostasis. Activation of innate immunity results in the induction of inflammation, which is essence, aims to restore the structural and functional integrity of tissues and organs.

Adenosine 5^′^-triphosphate (ATP) is thought to be one of the DAMPs playing a major role in the inflammation and cytokine storm. It is present in high concentrations within the cytoplasm of every cell and can be released after cell damage by a variety of injurious agents [1,2]. After release, ATP acts through binding to specific receptors known as purinergic P2 receptors (P2R), of which the *P2X*_*7*_ subtype is a potent mediator of cytokine processing and release [3].

The purinergic *P2X*_*7*_ receptor is a ligand-gated ion channel which has a wide tissue distribution, being expressed by virtually all cell types, including cells of the immune system, i.e. monocytes, macrophages, dendritic cells, and T cells [4]. Activation of this receptor by brief exposure to extracellular ATP opens a cation channel, which allows Ca^2+^ influx, as well as K^+^ efflux [5]. Longer exposure to ATP leads to dilatation of the *P2X*_*7*_ channel to a pore, which allows uptake of permeants up to the size of ethidium^+^[6,7]. Activated *P2X*_*7*_ receptors are known to play an important role in regulating the inflammatory response *in vivo* (reviewed in [8]. Research indicates that activation of the P2X_7_ receptor causes massive release of the pro-inflammatory mediator IL-1β. The *P2X*_*7*_-mediated release of IL-1β by immune cells is suggested to be regulated by various mechanisms including: a) cytotoxicity of IL-1β producing immune cells [9], b) K^+^ efflux [9], and c) activation of the inflammasome NALP3 via pannexin-1 (reviewed in [10]). In addition to IL-1β, other pro-inflammatory mediators are also up-regulated via *P2X*_*7*_ receptor, including IL-6, IL-18 and TNF-α (reviewed in [11]).

These data point to an important role of P2X7 receptor-mediated signaling in inflammation, and also suggest that polymorphisms within the *P2X*_*7*_ receptor gene that lead to loss of receptor function have the potential to impair cytokine release by immune cells in vivo.

Several non-synonymous single nucleotide polymorphisms (SNP) have been characterized in the *P2X*_*7*_ receptor gene (reviewed in [12]). One such SNP concerns the nucleotide at position 1513, which changes a glutamic acid to an alanine acid at amino position 496 (*Glu496Ala*). Previous research in human monocytes, showed that the *Glu496Ala* polymorphism decreased the *P2X*_*7*_ receptor mediated K^+^ efflux, thereby delaying *P2X*_*7*_ receptor mediated release of IL-1β [13]. Furthermore, it was shown that subjects homozygous for the variant allele of the *Glu496Ala* polymorphism had reduced sensitivity to inflammation compared to wild-type subjects [14]. In the present study, we further tested the hypothesis that subjects homozygous for the *Glu496Ala* loss-of-function polymorphism produce lower levels of IL-1β in response to ATP. In addition to levels of IL-1β, we also explored whether production of other inflammatory cytokines in response to ATP was altered in subjects carrying the *Glu496Ala P2X*_*7*_ receptor SNP.

To test our hypotheses, we used an ex vivo inflammation model by stimulating whole blood with the potent inflammatory stimuli LPS and PHA (phytohemagglutinin). Previous research showed that this whole blood assay, in contrast to isolated cells or cell lines grown in medium, closely resembles the in vivo situation and forms an appropriate and reproducible culture condition to measure cytokine production ex vivo [15].

## Results

### Study population

Of the eligible 14 *P2X7MUT* subjects, a total of 9 *P2X7MUT* (aged 50-75 years; 2 men and 7 women)donated blood for this ex vivo experiment. Two out of these 9 *P2X7MUT* subjects showed no other SNPs in the *P2X*_*7*_ receptor gene. The other seven subjects were shown to have several other non-synonymous SNPs in the *P2X*_*7*_ receptor gene in addition to the *Glu496AlaP2X*_*7*_ receptor polymorphism (Table 1).

**Table 1:** **P2X_7_*genotype distribution for the study subjects (N=16)***

**Glu496Ala**	**NullAllele (151+1g>t) rs35933842**	**Val76Ala rs175 25809**	**Arg117Trp rs28360445**	**Gly150Arg rs283 60447**	**His155Tyr rs208294**	**Glu186Lys rs28360451**	**Leu191Pro rs28 360452**	**Null Allele (699C→T) n.a.**	**Arg270Cys rs16950860**	**Arg307Gln rs28360457**	**Ala348Thr rs1718119**	**Thr357Ser rs2230911**	**Gln460Arg rs2230912**	**Ile568Asn rs1653624**	**No. of subjects**
***HOMO***	WT	WT	WT	WT	WT	WT	WT	WT	WT	WT	WT	WT	WT	WT	2
***HOMO***	***HET***	WT	WT	WT	WT	WT	WT	WT	WT	WT	WT	WT	WT	WT	3
***HOMO***	WT	WT	WT	WT	***HET***	WT	WT	WT	WT	WT	WT	WT	WT	WT	2
***HOMO***	WT	WT	WT	***HET***	WT	WT	WT	WT	WT	WT	WT	WT	WT	WT	1
***HOMO***	WT	WT	WT	***HET***	WT	WT	WT	***HET***	WT	WT	WT	WT	WT	WT	1
WT	WT	WT	WT	WT	WT	WT	WT	WT	WT	WT	WT	WT	WT	WT	7

HOMO: Homozygous for the variant allele.

HET: Heterozygous.

WT: Wild-type.

Of the eligible 19 *P2X7WT* subjects, a total of 7 *P2X7WT* (aged 55-74 years; 1 man and 6 women) were willing to donate blood.

### Effects of LPS/PHA stimulation on cytokine release

Stimulation of whole blood from P2X_7_MUT subjects with LPS + PHA for 24 hours induced a strong rise in levels of IL-1β, TNF-α, IL-6, IL-10 and IFN-γ (Table 2).

**Table 2:** ***Effect of LPS + PHA stimulation on the release of cytokines *(Release of IL-1β, TNF-α, IL-6, IL-10 and IFN-γ in whole blood from wild type subjects (P2X7WT) and subjects homozygous for the Glu496Ala polymorphism (P2X7MUT)^a)^)**

	***Unstimulated***		***p-value***	***Stimulated***		***p-value***
	**P2X7WT**	**P2X7MUT**		**P2X7WT**	**P2X7MUT**	
**IL-1β**	2.2 ± 0.16	2.6 ± 0.35	0.381	5,644 ± 3,261	3,440 ± 1,647	0.529
**TNF-α**	6.6 ± 1.04	7.1 ± 0.33	0.694	7,784 ± 3,470	5,173 ± 1,380	0.504
**IL-6**	2.5 ± 0.21	2.5 ± 0.35	0.965	13,922 ± 5,790	19,804 ± 8,003	0.064
**IL-10**	2.4 ± 0.21	2.0 ± 0.10	0.190	291 ± 104	305 ± 167	0.968
**IFN-γ**	15.4 ± 3.93	13.4 ± 6.38	0.792	701 ± 283	791 ± 144	0.766

^a)^*Data are shown as mean ± SEM.*

No statistically significant differences in IL-1β, TNF-α, IL-6, IL-10 and IFN-γ levels in unstimulated whole blood in the absence of ATP were observed between *P2X7MUT* and *P2X7WT* subjects. In LPS/PHA-stimulated blood, levels of IL-1β and TNF-α tended to be slightly higher and levels of IL-6, IL-10 and IFN-γ tended to be slightly lower in LPS/PHA-stimulated whole blood from *P2X7MUT* subjects, even though the differences were not statistically significant (Table 2).

No effect of the *P2X*_*7*_ receptor on the production of the other measured cytokines (i.e. IL-2, IL-7, IL-8, IL-12, IL-13, IL-17, G-CSF, GM-CSF, MCP-1, MIP1-β) was observed (data not shown).

### Effect of the Glu496Ala polymorphism on IL-1β release in response to ATP

**Figure 1:**
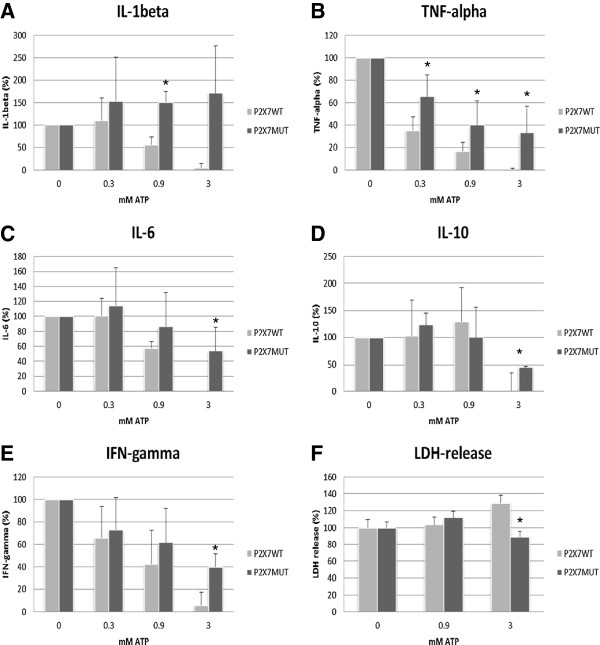
***Cytokine and LDH release *A-E LPS/PHA-induced cytokine release in response to 24 h stimulation by ATP at different concentrations (0.3, 0.9 and 3 mM), in subjects without the Glu496Ala P2X_7_ receptor polymorphism (P2X7WT) vs. subjects with this SNP (P2X7MUT).** Results are expressed as percent change from baseline (LPS/PHA-stimulation without ATP incubation) ± SEM. **E** LDH release in response to ATP stimulation in subjects without the Glu496Ala P2X_7_ receptor polymorphism (P2X7WT) vs. subjects with this SNP (P2X7MUT) * Significant difference between P2X7MUT and P2X7WT (p < 0.05).

The effect of ATP at different concentrations on LPS/PHA–induced IL-1β release in whole blood after 24hours is shown in Figure 1A. ATP at a concentration of 0.3mMhad no effect on the LPS/PHA-induced IL-1β release among *P2X7WT* subjects, whereas IL-1β levels in blood from *P2X7MUT* subjects were increased to 160% of levels at 0 mM ATP. At higher ATP concentration IL-1β levels in blood from *P2X7WT* subjects were reduced (i.e. 57% and 4.7% of baseline levels at 0.9mM and 3mM ATP respectively), whereas *P2X7MUT* subjects showed increased levels of IL-1β (i.e. 151% and 173% of baseline levels at 0.9mM and 3mM ATP respectively) The decreased IL-1β levels observed at the ATP concentration of 0.9mM was consistent in 6 out of the 7 *P2X7WT* subjects, and the almost complete abolishment of IL-1β production in response to 3.0mM ATP was consistent in all 7 *P2X7WT* subjects.

When comparing *P2X7WT* with *P2X7MUT* subjects, the IL-1β response of *P2X7WT* subjects, to ATP at 0.9 and 3 mM was only 65% (p=0.031) and 3% (p=0.058), respectively, of the IL-1β response to ATP observed in *P2X7MUT* subjects.

### Effects of the Glu496Ala polymorphism on TNF-α,IL-6, IL-10 and IFN-γ release in response to ATP

Effects of 24-hour LPS/PHA stimulation of whole blood in the presence of ATP at different concentrations on the release of TNF*-*α, IL-6, IL-10 and IFN-γ are shown in Figure 1B-E. Figure 1B shows that in both *P2X7WT* and *P2X7MUT* subjects, levels of TNF*-*α decreased after incubation with ATP. This effect was consistent in all studied subjects. Comparing *P2X7WT* with *P2X7MUT* subjects, we observed a significantly stronger reduction in TNF*-*α levels relative to baseline in *P2X7WT* subjects (*P2X7MUT* vs. *P2X7WT*: 36% vs. 64% (p=0.008), 60% vs. 83% (0.041), and 67% vs. 99% (p=0.011) at 0.3 mM, 0.9 mM and 3 mM ATP, respectively).

Figure 1C shows that ATP at a concentration of 0.3mMhad no effect on the LPS/PHA-induced IL-6 release among *P2X7WT* subjects, whereas a slight increase in IL-6 release to 114% of levels with 0 mM ATP was observed in *P2X7MUT* subjects. Both *P2X7WT* and *P2X7MUT* subjects showed a decline in IL-6 release at 0.9 mM and 3.0 mM ATP compared to baseline. This inhibition of IL-6 release, however, was less pronounced in *P2X7MUT* subjects (*P2X7WT:*42% and 100%; *P2X7MUT*14% and 46%, respectively). The difference in IL-6 release between *P2X7WT* and *P2X7MUT* subjects was significant at 3 mM (p=0.050).

*P2X7WT* subjects showed no alteration in the IL-10 release in response to 0.3 mM ATP compared to baseline levels, and increased levels of IL-10 were observed at an ATP concentration of 0.9 mM (129%). Again, at 3 mM ATP, the release of IL-10 in blood from *P2X7WT* subjects was almost completely abolished, i.e. a 99% reduction in IL-10 levels compared to baseline levels. In *P2X7MUT* subjects, 0.3 mM ATP induced an increase in LPS/PHA-induced IL-10 release to 122% of baseline levels (Figure 1D), whereas at ATP concentrations of 3 mM the release of IL-10 in *P2X7MUT* subjects declined to 45% of baseline levels. These levels were significantly higher than IL-10 levels in blood from *P2X7WT* subjects at 3 mM ATP.

At an ATP concentration of 0.3mM, a 34% reduction of IFN-γ production compared to baseline was observed in *P2X7WT* subjects (Figure 1E). A further reduction in IFN-γ production was found at 0.9 and 3 mM ATP; 58% and 94%, respectively. In *P2X7MUT* subjects also reduced IFN-γ levels were observed at all ATP concentrations compared to baseline (27%, 39% and 60%, respectively).

### ATP measurement in whole blood

No difference in ATP levels were observed between P2X7MUT and P2X7WT subjects.

### LDH release

At an ATP concentration of 0.9mM, the release of LDH showed a slight increase compared to baseline in *P2X7WT* and *P2X7MUT* subjects, to 103% and 112% of levels without ATP, respectively (Figure 1F). *P2X7WT* subjects showed a further increase in the release of LDH at ATP concentration of 3 mM to 135% of the condition without ATP, whereas a decrease in the release of LDH to 95% of the condition without ATP was observed in *P2X7MUT* subjects. The difference between the change in *P2X7MUT* and *P2X7WT* subjects at 3mM ATP was statistically significant (p=0.014).

## Discussion

In vitro evidence suggests that the *P2X7* receptor is involved in the induction of inflammation. In the present study, we tested the hypothesis that *P2X7MUT* subjects have impaired cytokine release compared to *P2X7WT* subjects, using an ex vivo inflammation model. In this model, blood was stimulated with the potent inflammatory stimuli LPS and PHA to trigger an inflammatory response via both the innate and adaptive arms of immunity, respectively, leading to the production of a range of different cytokines. Our group previously showed that stimulatory effects of low-level ATP (i.e. 0.3 mM) on the production of IL-10 were maximal after 24 hours of incubation, and that early inhibitory effects of 0.3 mM ATP on IL-1β, TNF-α, IL-6 and IFN-γ persisted up to this time point [16]. In the present study, whole blood from subjects with and without the *Glu496Ala* polymorphism in the *P2X*_*7*_ receptor gene were incubated with ATP concentrations ranging from 0.3 to 3 mM to evaluate effects of this loss-of-function polymorphism on cytokine release via *P2X*_*7*_.

Contrary to our hypothesis, LPS/PHA-induced IL-1β release showed a concentration-dependent stimulation by ATP incubation of blood from *P2X7MUT* subjects, whereas ATP induced a concentration-dependent attenuation of the LPS/PHA-induced IL-1β release in *P2X7WT* subjects. However, the most striking result observed in the present study was that release of IL-1β as well as release of TNF*-*α, IL-6, IL-10 and IFN-γ was almost completely abolished in whole blood from *P2X7WT* subjects after 24-hour LPS/PHA stimulation in the presence of 3 mM ATP, whereas such an effect was not observed in *P2X7MUT* subjects.

It is known that apoptosis, which is a morphologically distinct form of cell death [17], is induced in immune cells by prolonged or excessive activation of the *P2X*_*7*_ receptor by high levels of ATP [18]. Since impaired *P2X*_*7*_ receptor function in leucocytes from subjects with the *Glu496Ala* polymorphism could lead to resistance to *P2X*_*7*_-mediated apoptosis, we measured LDH levels as an indicator of ATP-induced cell death in the present study. Results showed increased LDH levels after 24-hour exposure to high concentrations of ATP in *P2X7WT* subjects, but not in *P2X7MUT* subjects. This finding, combined with the striking observation that levels of all cytokines were almost completely abolished in*P2X7WT* subjects after 24-hour LPS/PHA stimulation of whole blood in the presence of 3 mM ATP, would suggest that leucocytes from *P2X7MUT* subjects were protected against ATP-induced cell death, presumably via impaired *P2X*_*7*_-mediated apoptosis. This result is in line with a previous study, which showed a marked reduction in non-viable lymphocytes in *P2X7MUT* subjects [7], strengthening the hypothesis that the *Glu496Ala* polymorphism indeed causes resistance to apoptosis.

In studies in isolated cell lines, it has been well established that extra cellular ATP at high concentrations triggers massive release of IL-1β, thereby inducing a strong pro-inflammatory response [19,20]. Numerous *in vitro* studies as well as *in vivo* studies with *P2X*_*7*_ receptor knock-out mice have demonstrated that the *P2X*_*7*_ receptor is the main receptor responsible for the release of IL-1β induced by ATP [21-23]. Therefore, polymorphisms is the P2X7 receptor gene causing a function change in the *P2X*_*7*_ receptor, such as the *Glu496Ala* polymorphism, could lead to impaired IL-1β releases. Sluyter et al. [13] previously showed that in whole blood from subjects harbouring the *Glu496Ala* polymorphism IL-1β release was 78% lower than in whole blood from wild-type subjects after 30 min of 6 mM ATP treatment. However, after 60 min ATP treatment this inhibition of IL-1β release was no longer apparent [13]. More recently, also a 6-hour incubation with 1mM ATP did not induce an attenuation of IL-1β release in *P2X7MUT* subjects [14]. It has been demonstrated that the *Glu496Ala* polymorphism results in impaired ATP-induced pore formation, but does not cause a total loss of *P2X*_*7*_ channel function [7]. Indeed, Sluyter et al [24] found that *P2X7MUT* subjects showed a reduced but not totally abolished *P2X*_*7*_ receptor-mediated K^+^ efflux compared to *P2X7WT* subjects. The authors suggested that the smaller residual K^+^ efflux may still be sufficient to cause delayed release of IL-1β in subjects harbouring the *Glu496Ala* polymorphism, offering a putative explanation why no reduction in IL-1β release has been observed after prolonged incubation with ATP in monocytes from these subjects. In our study, we observed even increased levels of IL-1β in *P2X7MUT* subjects compared to *P2X7WT* subjects after 24-hour ATP incubation. This result might be explained by the fact that the *Glu496Ala* SNP incompletely impairs *P2X*_*7*_ receptor function, that is, delaying but not abolishing the K^+^ efflux that is essential for IL-1β release, while impairing pore formation is essential for apoptosis. Another possible explanation for the observed increased IL-1β release in *P2X7MUT* subjects might be the activation of immune cells by other proinflammatory cytokines, such as TNF-α and IFN-γ[25,26]. It is difficult, however, to draw conclusions about differences between *P2X7WT* vs. *P2X7MUT* subjects regarding effects of ATP at 3 mM on production of IL-1β as well as production of other cytokines, since the effects of ATP at these concentrations in our study appeared to be distorted by cytotoxic effects via *P2X*_*7*_ in *P2X*_*7*_ WT subjects.

Previous findings showed the involvement of the *P2X*_*7*_ receptor in the ATP-induced secretion of IL-6 in several diseases [27]. In the present study, however, decreased levels of IL-6 were observed after incubation of LPS/PHA stimulated blood with ATP. Furthermore, this decrease was less pronounced in *P2X7MUT* subjects compared to *P2X7WT* subjects. Future research on the effects of ATP induced release of IL-6, and the involvement of the *P2X*_*7*_ receptor in IL-6 release is therefore warranted.

In a previous study by our group in which we used the whole blood assay to test the influence of ATP on the release of a number of cytokines [16], it was shown that co-incubation of whole blood from healthy volunteers with LPS/PHA and 0.3 mM ATP attenuated the rise of the pro-inflammatory cytokine TNF-α and stimulated the secretion of the anti-inflammatory cytokine IL-10. We demonstrated pharmacologically [28] that the stimulation of TNF-α release and the inhibition of IL-10 release were due to ATP-induced activation of the P2Y_11_and P2Y_12_ receptor subtypes, respectively. In agreement with these earlier studies, we found in the present study that ATP at 0.3 mM decreased TNF-α levels in both *P2X7WT* and *P2X7MUT* subjects. Although pharmacological evidence in rat microglia suggests that TNF-α levels may be increased via activation of the *P2X*_*7*_ receptor [29], our data would rather suggest that the *P2X*_*7*_ receptor is not involved in the regulation of TNF-α release in whole blood ex vivo at low levels of ATP.

Little is known about the direct regulation of production of IL-10 by the *P2X*_*7*_ receptor. Denlinger et al. [14] showed that P2X7MUT subjects released higher levels of IL-10 in response to ATP than *P2X7WT* subjects. Our data, however, do not show a differential effect of ATP at 0.3 and 0.9mM on IL-10 in *P2X7MUT* vs. *P2X7WT* subjects.

It is known that hemolysis can significantly increase plasma ATP concentration [30]. Therefore, the differences between P2X7MUT and P2X7 WT subjects found in the present study could have been confounded by the different amount of hemolysis in the samples of these subjects. However, our HPLC data shows that the plasma ATP levels of P2X7MUT and P2X7 WT were similar and are unlikely to have influenced our results.

In this study we only focused on the role of the P2X7 receptor subtype in the inflammatory response. Several other members of the P2 purinergic receptor family have been shown to play a in the ATP induced inflammatory response [31]. Moreover, recent evidence has indicated a role for the P2X4 receptor subtype in the regulation of P2X7-mediated inflammatory functions [32]. Therefore, our results might have been influenced by the presence of non-synonymous SNPs within other P2X receptor genes causing functional changes of these receptors. In the body, an interplay exists between bone homeostasis and immune processes. Research dealing with crosstalk between the bone and immune system, i.e. the field of osteoimmunology, is receiving growing attention [33,34]. Bone marrow is the principal site of haematopoiesis allowing a close interaction between bone and immune cells. Cytokines released during immune responses are known to affect the bone metabolism, and dysregulated cytokine responses are implicated in bone disease such as osteoporosis. IL-1β as well as TNF-α and IL-6 are known to promote osteoclastogenesis either by increasing osteoclast generation and activation or by inducing RANKL expression by osteoblasts [35,36], whereas IL-10 and IFN-γ are known to be inhibitors of osteoclastogenesis by blocking RANKL signalling, either directly or indirectly [35]. Alterations in cytokine release due to the *Glu496Ala* polymorphisms leading to loss of *P2X*_*7*_ receptor function might therefore contribute to the development of osteoporosis. As we observed increased levels of both IL-1β and TNF-α in *P2X7MUT* subjects relative to *P2X7WT* subjects, it might be suggested that *P2X7MUT* subjects have an increased risk to develop osteoporosis. This hypothesis is consistent with previous reports showing that the *Glu496Ala* polymorphism, leading to a non-functional *P2X*_*7*_ receptor, is associated with decreased BMD values, i.e. increased osteoporosis risk [37-39].

## Conclusions

In conclusion, a difference in the ATP-induced cytokine release and cell-death was observed between *P2X7MUT* subjects and *P2X7WT* subjects, indicating that the *Glu496Ala* loss-of-function polymorphism is likely to play an important during inflammation. Therefore, the P2X7 receptor might be of importance in the aetiology and pathophysiology of inflammatory diseases as well as related conditions, such as osteoporosis. However, further research to unravel the exact role of non-synonymous SNPs within the *P2X*_*7*_ receptor gene is warranted.

## Methods

### Study population and design

For our ex vivo experiment, we recruited participants from a previously established cohort in which *P2X*_*7*_ receptor SNPs have been genotyped. This cohort consisted of 376 men and women aged ≥ 50 years with a recent fracture. Subjects selected for the present study were previously identified either as wild type for the *Glu496Ala* polymorphism, or homozygous for the Glu496Ala polymorphism. Of the 376 subjects, 14 were homozygous for the *Glu496Ala* polymorphism(*P2X7MUT*), and 19 were wild-type for the *Glu496Ala* polymorphism (*P2X7WT*) and at the same time for all other genotyped *P2X*_*7*_ receptor SNPs [37].

All 33 selected subjects were contacted to give extensive oral information about the present study. After obtaining written informed consent, heparinized blood was obtained. Blood from fasting subjects was collected in two 9-ml heparin tubes between 8:30 and 9:30 AM on the morning of the whole blood experiment.

The study was approved by the ethical committee of the University Hospital Maastricht and Maastricht University.

### Blood-based cytokine production assay

The whole blood assay was performed according to the procedures described by Swennen and co-workers[16,40]. Briefly, concentrations of 17 cytokines (i.e. mature IL-1β, IL-2, IL-4, IL-5, IL-6, IL-7, IL-8, IL-10, IL-12, IL-13, IL-17, G-CSF, GM-CSF, IFN-γ, MCP-1, MIP1-β, TNF-α) were determined in heparinized blood samples, either unstimulated or stimulated with 10 μg/ml LPS (lipopolysaccharide) and 1 μg/ml PHA (phytohemagglutinin) to induce an inflammatory reaction, and incubated with different concentrations of ATP (0, 0.3, 0.9, and 3 mM) for 24 hours, by using Luminex Multiplex X-map technology (Bio-Rad Laboratories B.V., Veenendaal, the Netherlands).

### Measurement of ATP by luminescence

Samples from the whole blood assay were collected at different time points following incubation (t=0, 30min and 2, 4, 6, 24 h) for measurement of ATP levels by luminescence. Samples were diluted with 50μl Fire Zyme Dilution Buffer (Bio-Medica Diagnostics, Inc., Windsor, NS, Canada) and immediately centrifuged at 3000g for 5 min at 4°C. A Cell Titer-Glow ATP-assay kit (Promega Benelux BV, The Hague, The Netherlands) was used according to the manufacturer’s instructions to measure the bioluminescence on a Glomax plate reader.

### Lactate dehydrogenase measurement

To measure cytotoxicity, lactate dehydrogenase (LDH) leakage was estimated. Although this necrotic cell death marker does not directly measure ATP-induced cell apoptosis, it is considered a useful marker of apoptosis, since apoptosis is a transient process leading to secondary necrosis [41].

For this, 50 μl cell-free supernatant samples from the whole blood assay were transferred into 96-well plates and LDH was measured using the CytoTox96 nonradioactive cytotoxicity assay kit (Promega Benelux BV, The Hague, The Netherlands). LDH release at ATP concentrations of 0.9 and 3mM is expressed as percentage difference relative to LDH release in LPS/PHA stimulated samples without ATP incubation. Since the half maximal concentration of the P2X_7_ receptor is approximately 0.5mM, concentrations of 0.3mM ATP were not measured.

### Statistical analysis

Results are reported as mean and standard error of the mean (SEM). Differences in cytokine release between *P2X7MUT* and *P2X7WT* subjects were compared using independent samples T-tests, after testing for normality of the distribution. P-values equal to 0.05 or below, were regarded as statistically significant. All analyses were performed using SAS, version 9.1.

## Competing interests

The authors declare that they have no competing interests.

## Authors' contributions

AW and ET carried out the experiment. AW and MB analyzed data and performed the statistical analysis. I. Arts, PD and PG conceived of the study, and participated in its design and coordination and helped to draft the manuscript. All authors read and approved the final manuscript.
